# The Effect of Calf Gender on Milk Production in Seasonal Calving Cows and Its Impact on Genetic Evaluations

**DOI:** 10.1371/journal.pone.0151236

**Published:** 2016-03-14

**Authors:** Melanie K. Hess, Andrew S. Hess, Dorian J. Garrick

**Affiliations:** 1 Department of Animal Science, Iowa State University, Ames, Iowa, United States of America; 2 Livestock Improvement Corporation, Hamilton, New Zealand; 3 Institute of Veterinary, Animal and Biomedical Sciences, Massey University, Palmerston North, New Zealand; University of Tasmania, AUSTRALIA

## Abstract

Gender of the calf whose birth initiates lactation could influence whole lactation milk yield of the dam due to hormonal influences on mammary gland development, or through calf gender effects on gestation length. Fetal gender could influence late lactation yields because cows become pregnant at peak lactation. The effects of calf gender sequences in parities 1–3 were assessed by separately fitting animal models to datasets from New Zealand comprising 274 000 Holstein Friesian and 85 000 Jersey cows, decreasing to 12 000 and 4 000 cows by parity 3. The lactation initiated by the birth of a female rather than a male calf was associated with a 0.33–1.1% (p≤0.05) higher milk yield. Female calf gender had carryover effects associated with higher milk yield in second lactations for Holstein Friesians (0.24%; p = 0.01) and third lactations for Jerseys (1.1%; p = 0.01). Cows giving birth to bull calves have 2 day longer gestations, which reduces lactation length in seasonal calving herds. Adding a covariate for lactation length to the animal model eroded some of these calf gender effects, such that calving a female led to higher milk yield only for second lactation Holstein Friesians (1.6%; p = 0.002). The interval centering method generates lower estimates of whole lactation yield when Wood’s lactation curves are shifted to the right by 2 days for male calves and this explained the higher yield in female calves when differences in lactation length were considered. Correlations of estimated breeding values between models including or excluding calf gender sequence were 1.00 for bulls or cows. Calf gender primarily influences milk yield through increased gestation length of male calves, and bias associated with the interval centering method used to estimate whole lactation milk yields. Including information on calf gender is unlikely to have an effect on selection response in New Zealand dairy cattle.

## Introduction

Dairy farm profitability is influenced by milk income minus animal-related costs. Milk income is associated with milk yield and composition, which are influenced by a number of environmental factors, as well as genetics. Offspring gender has been shown to affect milk yield in a number of species [1, 2], however it is often difficult to determine whether this effect is due to gestational differences or differences in suckling habits of the offspring [3]. *Bos taurus* dairy calves are typically weaned within hours of birth and artificially reared, allowing gestational differences to be un-confounded with suckling. Dairy cattle are commonly managed in large contemporary groups, enabling environmental factors to be accounted for more accurately, increasing statistical power.

Hormones produced by the bovine fetus are able to cross the placenta, and calf gender has been shown to impact hormonal levels in the dam [4]. Differences in the abundance of hormones involved in lactogenesis may influence milk yields in dams that give birth to male or female calves. The gender of the first parity calf may have an effect on milk yield in all later lactations if differences in abundance of a hormone influences mammary development [5], because dairy cows are first bred before they are fully mature. Milk yield in any particular lactation could be affected by the gender of the calf whose birth initiates lactation, or the gender of the calf *in utero*, because cows become pregnant with their next calf around peak lactation.

Calf gender has been shown to affect milk yield in U.S. [6], Canadian [7], French [8], Iranian [9] and Danish [10] dairy cattle populations. Hinde et al. [6] showed that U.S. Holstein dams that gave birth to daughters had higher milk yields than dams that gave birth to sons, and that first parity calf gender affected milk yield in subsequent lactations. The Canadian [7] and Iranian [9] studies also observed higher milk yields in Holstein dams that gave birth to a daughter rather than a son; however the effects were larger than those observed in U.S. Holsteins in the Iranian study and smaller in the Canadian study. First and second parity calf gender influenced milk yield in both first and second lactations in the Canadian study [7]. In contrast, the French [8] and Danish [10] studies found a very small increase in milk yield in both Holstein and Montbéliarde dams that gave birth to a son. The French and Canadian studies concluded that although there may be some effect of calf gender on dam milk yield, it was not large enough to impact profit [8] or encourage use of sexed semen [7]. However, the size of the effect in the Iranian study led to the conclusion that the effect of calf gender on milk yield needs to be accounted for when considering the use of sexed semen [9].

The New Zealand dairy industry differs from North American and French industries in that it is primarily pastoral, with cows living outdoors year-round and receiving limited feed supplements. New Zealand dairy cattle are seasonally mated to calve annually in the Spring. Cows pregnant with female calves have shorter gestation lengths than cows pregnant with male calves, resulting in their calving approximately 2 days earlier [11]. This 2 day earlier calving could result in a higher milk yield from dams that give birth to female calves due to a longer lactation since whole herds typically cease lactation on the same date.

Although studies have investigated the effect of calf gender on dam milk yield and the impact of this effect on sexed semen use, none have evaluated the impact of a calf gender effect on milk yield breeding value estimation. If a calf gender effect was present in a population, selection of bull mothers and progeny tested bulls may be biased due to the sex of their offspring. Genetic progress towards more profitable cows could be greater if this calf-gender bias was accounted for in breeding value estimation.

The objectives of this study using NZ data were to: 1) determine whether calf gender has an impact on total milk yield in Holstein Friesian and Jersey cows, and 2) evaluate whether inclusion of calf gender in breeding value estimation models is likely to increase response to selection.

## Materials and Methods

Lactation and calving records from 1995 until 2005 were obtained from the New Zealand Dairy Core Database through Livestock Improvement Corporation (LIC) for Holstein Friesian and Jersey dairy cows. No sexed semen was used during this period in New Zealand. Data was obtained from an existing database and therefore its use was not subject to ethics approval. The interval centering method [12], which uses at least four herd tests across a lactation, was used to calculate milk yield. Only cows whose first three calvings were between July and October in consecutive years with a single, live calf born without assistance were considered for use in this study. Lactation records were discarded if lactation length was unusually short (<100 days) or extremely long (>305 days). Cows had to have calf gender recorded for parities 1 through 3 for use in analysis of lactations 1 and 2; lactation 3 analyses required the cow to also have calf gender recorded for parity 4. The number of animals remaining after these criteria were applied can be found in Table 1. Reduction in the number of animals analyzed for lactation 2 compared to lactation 1 were largely caused by cows lacking recorded lactation length in lactation 2, while reduction in the number of animals analyzed for lactation 3 compared to lactation 2 were also influenced by the absence of recorded calf gender in the fourth parity.

**Table 1:** Number of Animals After Data Filtering.

Lactation	Holstein Friesian	Jersey
1	274 401	85 774
2	117 233	84 960
3	12 347	4 197

The effect of calf gender across parities was evaluated using the sequences of calf genders. Calf gender sequences for fitting in analyses of lactations 1 and 2 consisted of all 2^3^ = 8 sequences of male and female calves across the first three parities such as three males (MMM) or three females (FFF). Calf gender sequences for lactation 3 consisted of all 2^3^ = 8 sequences of male and female calves across parities 2–4; except for model 1 which used all 2^4^ = 16 sequences of male and female calves across the first four parities. Significance of calf gender sequences were determined based on t-statistics using comparison-wise α = 0.05.

### Single Lactation Milk Yield

An animal model was fit to estimate the effect of calf gender sequence on total milk yield in lactations 1–3 separately:

$$Y_{ijk}=CG_{i}+HY_{j}+A_{k}+e_{ijk}$$ (1)

The dependent variable (Y_ijk_) was total milk yield for the lactation of interest determined from individual test-day records using the interval-centering method. The fixed effects were CG_i_, the calf gender sequence and HY_j_, the combination of herd and year of the lactation record. The random effect of animal, A_k_, was assumed to have a variance-covariance proportional to the additive relationship matrix generated from a 6-generation pedigree, and e_ijk_ was the residual. The effects for each calf gender sequence were calculated in ASReml [13] and linear contrasts among the sequences were constructed to estimate the effect of calf gender in each parity. A simpler fixed effects model, excluding A_k_, was fitted for comparison with Hinde et al. [6]. Third parity calf gender was included in the calf gender sequence for analysis of first lactation milk yield despite there being no biological explanation for the effect of third parity calf gender on first lactation milk yield because if an effect was observed, it would indicate that there may be inadequacies with the model.

### Lactation Length

Bull calves, on average, have a longer gestation length than heifer calves [11]. A repeatability model was fit to quantify the effect of calf gender on lactation length in each lactation. Lactation length must have been recorded for at least two of the first three lactations for each cow. The model, fitted to 67 403 Holstein Friesian and 58 318 Jersey cows separately, was:

$$LL_{ijkl}=C_{i}+HY_{j}+A_{k}+E_{l}+e_{ijkl}$$ (2)

The dependent variable (LL_ijkl_) was the lactation length represented by the number of days in milk for that lactation. Fixed effects of C_i_, the gender of the calf that initiates that lactation and HY_j_ were fitted. The random effects were E_l_−the permanent environmental effect across lactations for an animal, and A_k_ and e_ijkl_ as described in Model 1.

### Milk Yield with Lactation Length

Model 1 was expanded to include the interaction between lactation length, as a covariate, and the gender of the calf that initiated that lactation to assess the extent to which lactation length explains the observed effect of CG.

$$Y_{ijklm}=LL_{i}.C_{j}+CG_{k}+HY_{l}+A_{m}+e_{ijklm}$$ (3)

The dependent variable (Y_ijk_) was total milk yield for the lactation of interest. Lactation length (LL_i_) and calf gender (C_j_) were as described in Model 2. The remaining variables were as described in Model 1.

### Artefacts Present in Total Lactation Milk Yield

Total lactation milk yield is not typically observed but is predicted from milk yields measured on test days, often undertaken monthly or alternate-monthly. Those test day records can be analyzed using a test-day model [14], as in Barbat, Hinde; or can be pooled to provide estimates of total lactation yield, for example, using interval centering, with subsequent analyses being applied to the predicted total lactation yields. It is conceivable that interval centering could create artefacts or biases due to variation in days in milk at a given test day caused by differences in calving date.

A study was performed to determine whether the interval-centering method gives biased results if lactation curves vary systematically due to differences in calving date. Herd tests take place for all cows in the herd on a certain date, therefore cows that produced male calves will tend to have fewer days in milk on test day than cows that produced female calves. One approach to model the shape of lactation curves uses the Wood’s curve [12], which involves three parameters. These three parameters were estimated from actual monthly test day records and the estimated parameters were then used to simulate daily lactation yields. We sampled daily lactation yields corresponding to test days and applied interval-centering to quantify the extent of any bias.

The shape of lactation curves were based on a single herd with 50 first parity Holstein Friesian cows, where the last cow calved 44 days after the first to represent a typical calving spread in a pastoral spring calving herd. Separate Wood’s curves [15] were fitted for each cow to the 8–10 herd test records taken throughout the lactation using the package minpack.lm in R [16]. The model equation for the Wood’s curve is:

$$y(t)=at^{b}\exp^{-ct}+e(t)$$ (4)

whereby t is time in days since the start of lactation, y(t) is the test day milk yield on day t, a, b and c are parameters describing the curve, and e(t) is the residual on day t, with residuals assumed to be uncorrelated between days and between individuals.

Herd test dates were chosen to provide 5–6 herd tests over lactation, which satisfied the requirements of the interval-centering method as applied in New Zealand. Predicted milk yields on the 5–6 days corresponding to herd tests were obtained for each of 44 possible days of calving, for each of the 50 different Wood’s curves, to produce a total of 44×50 curves.

The interval centering method was then applied to each of the 5–6 predicted test day yields to generate a predicted whole lactation yield. For each of the 50 cows, the difference in milk yield was calculated between each of the 44 calving days (representing female calves) and the same cows calving 2 days later (representing male calves). These differences in estimated whole lactation yields were weighted by the proportion of animals in the herd that calved on each day in order to simulate any artifacts in whole lactation yield that result from the application of the interval centering method.

In pastoral circumstances it is usual to cease milking the entire herd approximately 60 days prior to the next calving. This results in shorter lactation lengths for those cows calving later in the season compared to cows calving earlier. Accordingly, the interval centering calculations described above were repeated to represent truncated rather than 305-day lactations.

### Effect on Breeding Values

Model 3 including calf gender, and Model 5 excluding calf gender were separately used to obtain EBVs for each animal:

$$Y_{ijk}=LL_{i}+HY_{j}+A_{k}+e_{ijk}$$ (5)

where all variables were as described in Model 3. The random animal effect for each animal (A) is the breeding value, and its prediction from the model is its EBV. Pearson and Spearman Rank Correlations between EBVs in Models 3 and 5 were calculated separately for cows and bulls for each breed. In addition, the top 100 females and top 30 males based on EBV were compared from each model for each lactation to assess the extent to which fitting calf gender affects milk yield EBVs in elite animals, since the purpose of evaluation is to identify the elite animals for use as parents of the next generation of bulls.

## Results

### Single Lactation Milk Yield

**Fig 1:**
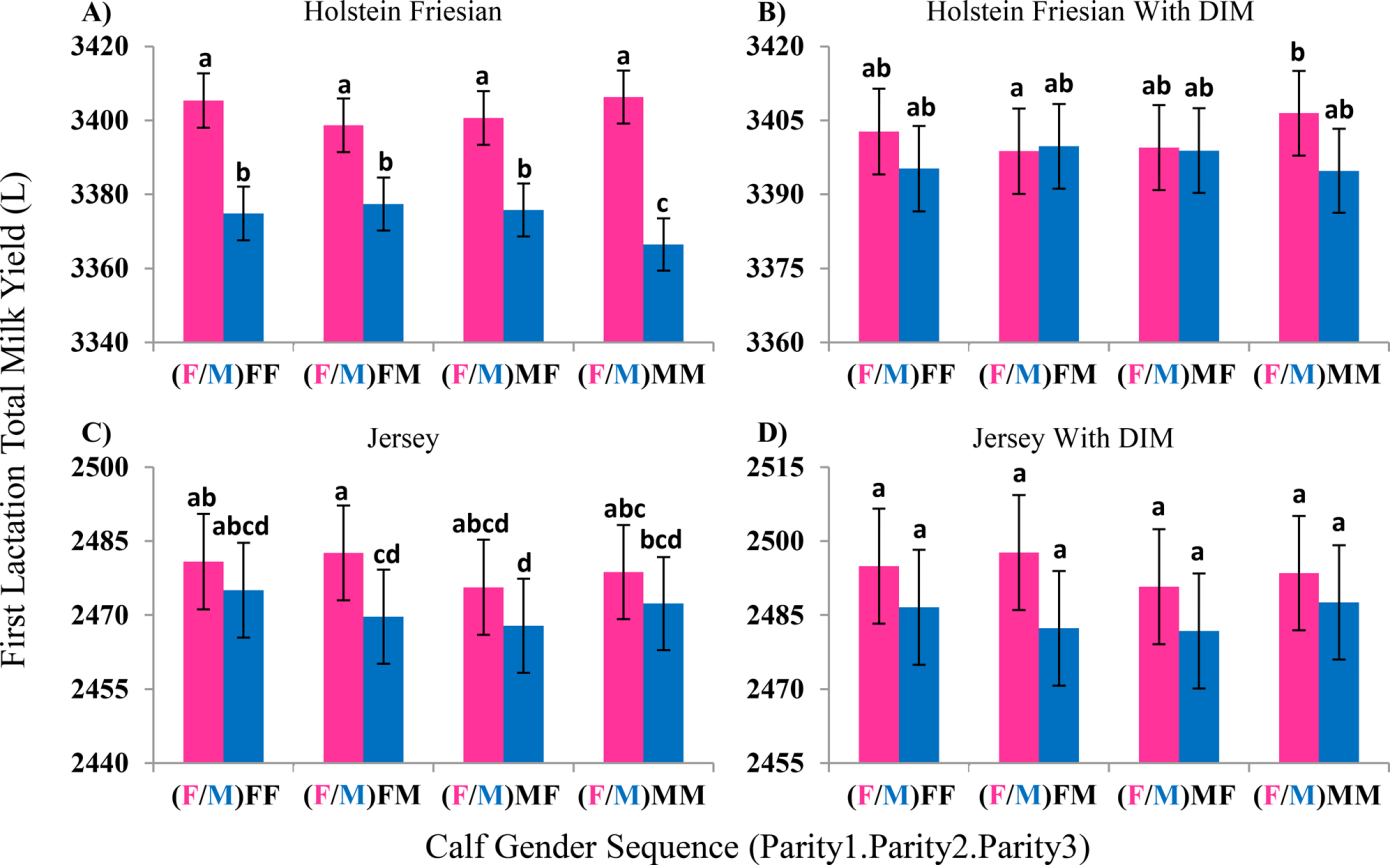
Effect of Parity One through Three Calf Gender Sequence on First Lactation Milk Yield. Analysis of 274,401 Holstein Friesian cows (A) showed that those who give birth to a female calf in their first parturition had higher first lactation milk yield than cows that had a male calf. Holstein Friesian cows with male calves at each of their first three parities had lower first lactation milk yield than other calf gender sequences. Fitting lactation length (DIM) as a covariate (B), decreased the difference between the calf gender sequences. Analysis of 85,774 Jersey cows (C) showed that having a female calf at first parturition was associated with higher first lactation milk yield. When lactation length (DIM) was added to the Jersey analysis (D) calf gender sequence retained the same trend as unadjusted data but differences were not significant. Bars with no letters in common in the same graph are significantly different from each other.

Cows that calved female calves in their first parity had higher first lactation milk yields in both Holstein Friesian (29 ± 2 L, p << 0.001) and Jersey (8 ± 3 L, p = 0.002) breeds. Calf gender in parities 2 or 3 did not influence first lactation milk yield (p ≥ 0.20); however, it was surprisingly found that Holstein Friesians cows that calved males in three consecutive years had significantly lower first lactation milk yield than any other calf gender sequence across the first three parities, including two males followed by a female (Fig 1A, p < 0.04). This finding is discussed further in the Discussion section. In Jerseys, the effects of calf gender were not as pronounced as in Holstein-Friesians and the numbers of observations were much fewer resulting in larger standard errors (Fig 1C).

**Fig 2:**
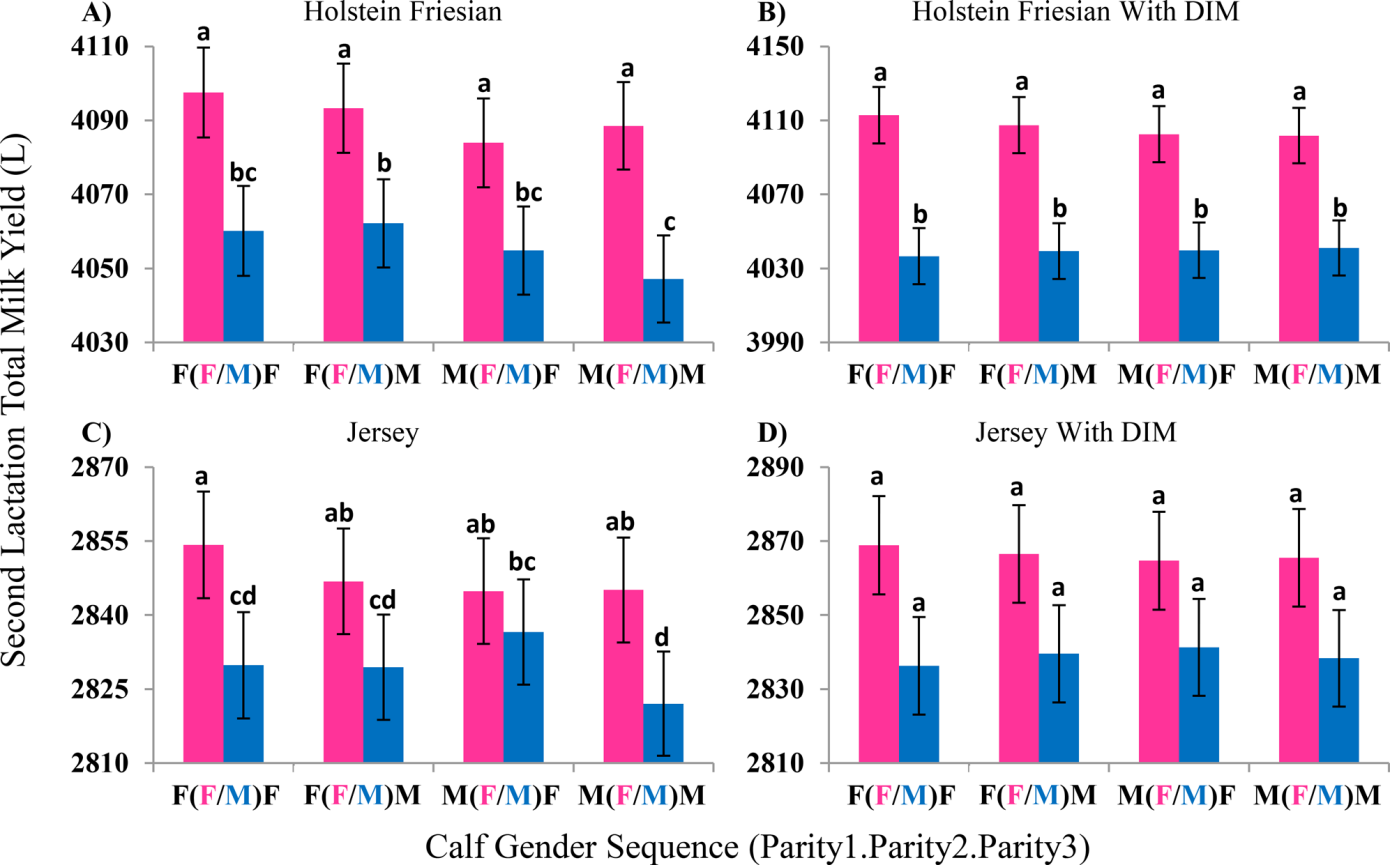
Effect of Parity One through Three Calf Gender Sequence on Second Lactation Milk Yield. Analysis of 117,232 Holstein Friesian cows (A) showed that second lactation milk yield was significantly higher if the calf born in the second parity was a female calf rather than a male calf. Holstein Friesian cows that gave birth to a female calf followed by two male calves had significantly higher milk yield than Holstein Friesian cows that gave birth to three males over the first three parities. When lactation length (DIM) was fit as a covariate in the analysis (B) only the second parity calf gender had a significant effect on second lactation milk yield. Analysis of 84,959 Jersey cows (C) showed that having three female calves in the first three parities gave significantly higher second lactation milk yield than having three male calves, but including lactation length (DIM) as a covariate in the analysis (D) showed the same trend as unadjusted data but differences were not significant. Bars with no letters in common in the same graph are significantly different from each other.

Cows with a female calf in the second parity had a higher second lactation milk yield in both Holstein Friesian (35 ± 4 L, p << 0.001) and Jersey (18 ± 3 L, p << 0.001) cows. In Holstein Friesians, a higher second lactation milk yield was also observed when giving birth to a female calf in the first parity (9 ± 4 L, p = 0.01). Interaction effects were identified between calf gender across the first three parities, with the lowest second parity milk yield observed when a cow gave birth to male calves in all three parities (Fig 2A and 2C).

First parity calf gender did not have a significant effect on third lactation milk yield in either Holstein Friesian or Jersey cows (p ≥ 0.3). That is, contrasts between effects for calf gender sequences that differed only by the calf gender in the first parity were not significantly different from each other (S1 Fig).

**Fig 3:**
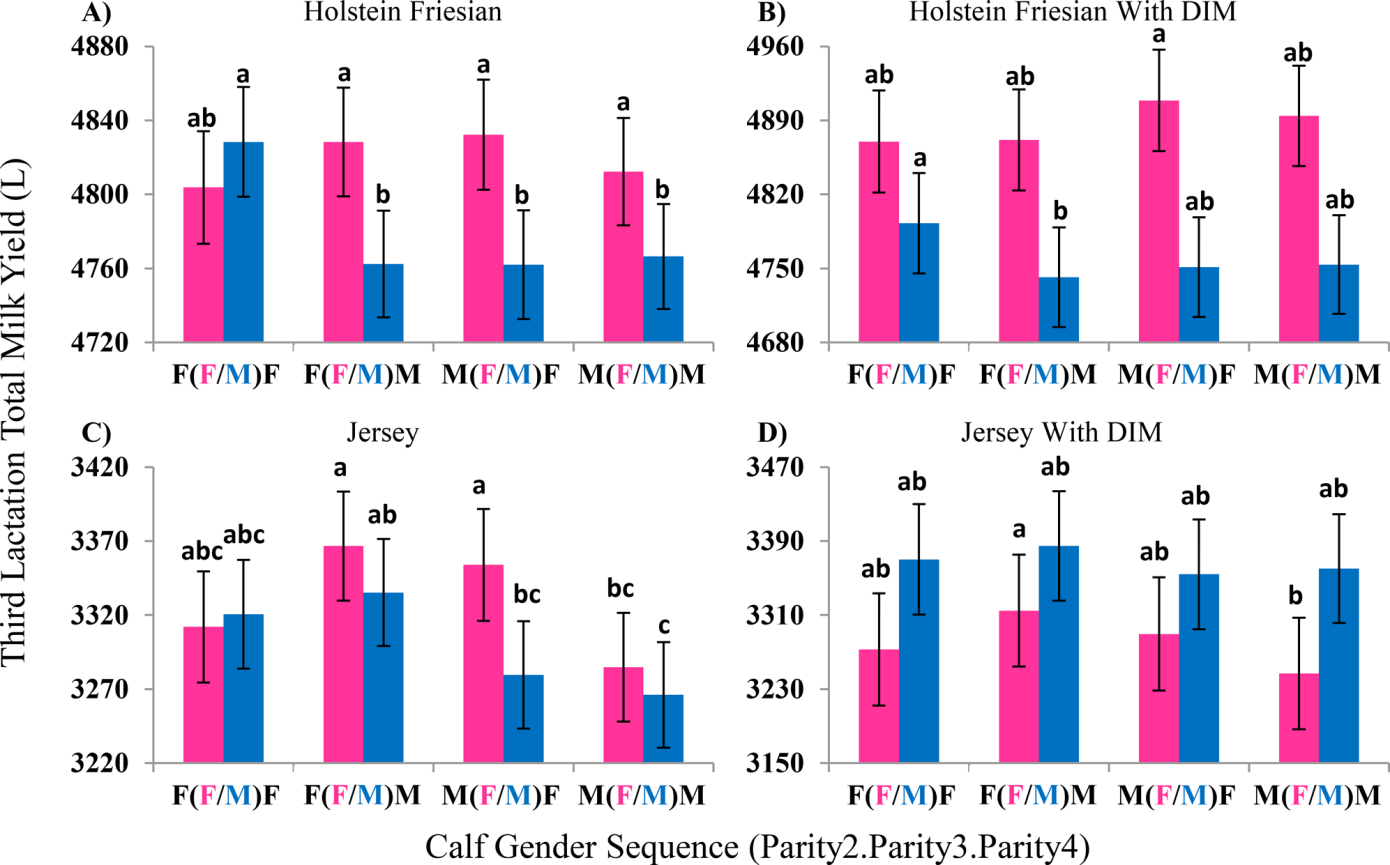
Effect of Parity Two through Four Calf Gender Sequence on Third Lactation Milk Yield. Analysis of 12,347 Holstein Friesian cows (A) showed that giving birth to a male calf in their third parturition resulted in lower milk yield than giving birth to a female calf in their third parturition, unless a female calf was born in the second and fourth parturition. When fitting lactation length (DIM) as a covariate (B), giving birth to a female in the third parturition was associated with higher milk yield than giving birth to a male calf. Analysis of 4,197 Jersey cows (C) showed cows that gave birth to a female calf in the second parity followed by male calves in the following two parities had significantly higher milk yield than cows that gave birth to male calves in each of parities two to four. When lactation length (DIM) was fit as a covariate (D), having a female in the second parturition tended to be associated with a lower milk yield than having a male calf. Bars with no letters in common in the same graph are significantly different from each other.

Holstein Friesian cows that gave birth to a female calf in the third parity had 39 ± 13 L (p = 0.002) higher third lactation milk yield compared to cows that gave birth to a male calf. No effect of third parity calf gender was observed in Jersey cows; however, cows that gave birth to a female calf in the second parity had 37 ± 15 L (p = 0.01) higher third lactation milk yield. Interaction effects were observed between the three-parity calf gender sequences in both breeds (Fig 3A and 3C) with no significant difference between third parity calf being male or female, when second and fourth calves are female, but all other scenarios resulted in higher third lactation yields associated with female calves compared to male calves.

Models excluding random animal effects with variance-covariance determined by pedigree relationships ignore correlations between residual effects that are due to half-sib and other relationships, but these did not change any of the findings.

### Lactation Length

The effect of calf gender on lactation length was assessed to determine whether lactation length may explain the observed difference in milk yield. The birth of a female calf resulted in a lactation between 1.1 and 3.2 days longer than if the calf were male, depending on the breed of the cow and lactation number (Table 2). These results suggest that some of the effect of calf gender on lactation yield may be explained by lactation length.

**Table 2:** Lactation Length in Days by Calf Gender within Breed and Lactation.

Breed	Lactation	Female Calf	Male Calf	Female—Male
		(Std. Err.)	(Std. Err.)	(Std. Err.)^1^
Holstein Friesian	1	235.8 (0.2)	234.1 (0.2)	1.6 (0.2)
Holstein Friesian	2	231.8 (0.2)	228.8 (0.2)	3.1 (0.2)
Holstein Friesian	3	229.3 (0.7)	226.9 (0.6)	2.4 (0.7)
Jersey	1	233.1 (0.3)	230.2 (0.3)	2.8 (0.5)
Jersey	2	235.4 (0.3)	234.3 (0.3)	1.1 (0.1)
Jersey	3	233.9 (0.6)	230.7 (0.6)	3.2 (0.7)

^1^Gender difference was significantly different from zero with p < 0.001 for all comparisons.

### Milk Yield with Lactation Length

Including a lactation length by first parity calf gender effect removed the significance of the effect of calf gender on first lactation milk yield in both Holstein Friesian (p ≥ 0.64) and Jersey (p ≥ 0.39) cows. Importantly, when the gender-specific lactation length effect was included in the model, having three males in each of the first three parities was no longer associated with a significantly lower first lactation milk yield in Holstein Friesian cows (Fig 1B). An interaction effect between the different levels of calf gender sequences was no longer observed in Jersey cows (Fig 1D). The regression of milk yield on lactation length was not significantly different for male and female calves in either Holstein Friesians (p = 0.84) or Jerseys (p = 0.39; Table 3).

**Table 3:** Regression Coefficients for Lactation Yield on Lactation Length in Liters per Day.

Breed	Lactation	Female Calf	Male Calf	Female—Male
	Number	(Std. Err.)	(Std. Err.)	(Std. Err.)
Holstein Friesian	1	13.50 (0.05)	13.51 (0.05)	0.01 (0.05)
Holstein Friesian	2	14.72 (0.10)	14.99 (0.09)	0.27 (0.10)*
Holstein Friesian	3	15.18 (0.33)	15.75 (0.30)	0.57 (0.33)
Jersey	1	9.40 (0.07)	9.46 (0.06)	0.06 (0.07)
Jersey	2	9.77 (0.08)	9.92 (0.07)	0.15 (0.08)*
Jersey	3	10.91 (0.40)	10.58 (0.36)	0.33 (0.40)

* Regression coefficients for males and females were significantly different α = 0.05.

Holstein Friesian cows that gave birth to a female calf in the second parity produced 62 ± 22 L (p = 0.006) more milk in their second lactation than cows that gave birth to a male calf when fitting a lactation length by second parity calf gender effect. A significant effect of second parity calf gender was not observed in Jersey cows (p = 0.13). The effects of first and third parity calf gender were not significant in either breed (p ≥ 0.43). There was no interaction between calf gender across parities (Fig 2B and 2D). The effect of lactation length on second lactation milk yield was different depending on whether a male or female calf is born in the second parity in both Holstein Friesians and Jerseys (p ≤ 0.04; Table 3).

Fitting a lactation length by third parity calf gender effect resulted in a reduction in the significance of the main effect of calf gender on third lactation milk yield in any parity for either Holstein Friesian or Jersey (p ≥ 0.08) cows. There were some significant interactions between calf gender across parities in both breeds, however more data is needed to fully explore these interactions (Fig 3B and 3D). The effect of lactation length was not dependent on whether a male or female calf was born in the third parity (p ≥ 0.08; Table 3).

### Artefacts Present in Total Lactation Milk Yield

**Fig 4:**
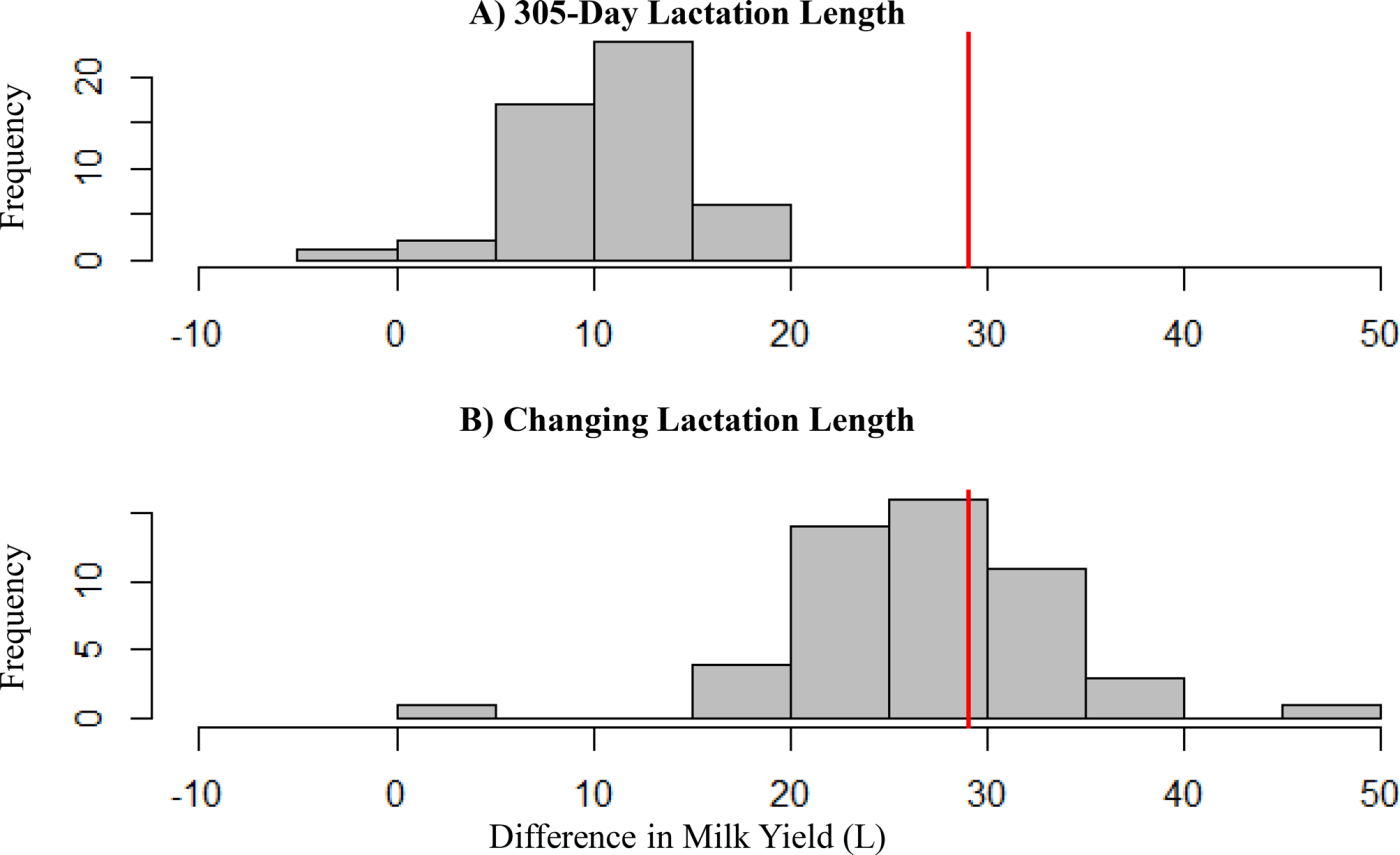
Effect of Delayed Calving of Male Calves With Interval-Centering Method. Herd tests that are consistently 2 days earlier in the lactation give total lactation milk yield between 2 L more and 18L less using the Interval-Centering method when lactation length is 305 days (A). In practice, a 2 day delay in calving leads to a 2 day shorter lactation. Graph B shows that the difference in total lactation milk yield when the difference in lactation length is taken into account is between 5 and 45 L less when calving is 2 days later. Vertical lines correspond to the observed difference in milk yield between a Holstein-Friesian cow that has a female calf compared to a male calf in parity 1 (red).

Milk yields for 305-day lactations based on fitted Wood’s curve (Fig 4A) were 10.8 ± 4.0 L higher for cows that were 2 days earlier in lactation on test days as would be expected for dams of shorter gestation female rather than male calves. The bias arising from interval-centering accounting for reduced lactation lengths for dams of male calves was even greater, representing differences in milk yield of 26.9 ± 6.2 L (Fig 4B).

### Effect on Breeding Values

Pearson and Spearman rank correlations for the sires were 1.00 in each lactation when considering all sires and also when only considering the top 30 sires for each breed. That is, the top 30 sires were the same, regardless of whether calf gender was included in the model, in lactation 1 in Jerseys. In the worst cases, there were still 29 of the top 30 sires in the model excluding calf gender also in the top 30 sires in the model including calf gender for lactations 2 and 3 in Jerseys and lactations 1–3 in Holstein Friesians. The sire that dropped out of the top 30 was ranked 30^th^ except for the third lactation in Jerseys where it was the 28^th^ ranked sire in the model without calf gender who was the 37^th^ ranked sire in the model with calf gender included. The slight re-ranking of animals did not change the mean EBV of the top 30 sires.

Pearson and Spearman rank correlations for cows were 1.00 when considering all animals and also when only considering the top 100 animals. The top 100 Holstein Friesian animals in the model excluding calf gender were also the top 100 animals in the model with calf gender included for lactations 1 and 2. At most four animals in the top 100 females in the model without calf gender dropped out of the top 100 in the model with calf gender for each of the other lactations. The largest drop among the top 100 dams when calf gender was added to the model, was an individual who was ranked 85^th^ and moved to 104^th^ in third lactation Jerseys. This individual was also the highest-ranked dam to drop from the top 100 dams in any breed or lactation. The re-ranking of animals had very little impact on the mean EBV of the top 100 dams.

## Discussion

### Model

Modeling of complex traits is challenging due to the number of biological and environmental factors that influence them. The statistical model used for analysis of complex traits can have a large effect on the results and the interpretation of the data. A number of recent studies have been published estimating the effect of calf gender on milk yield [6–10]. They each used a different model to evaluate this effect, although the Canadian study did not report the model used.

Hinde et al. [6] used an autoregressive (ARH1) covariance structure to model residual effects for multiple lactations on the same animal. The ARH1 covariance structure they fitted allows for heterogeneous variances at different lactations and assumes that the correlation between successive lactations, such as 1 and 2, or 4 and 5, is the same. The correlation between lactations separated by 2 parities such as 1 and 3, or 2 and 4 is the square of the correlation between successive parities, and those separated by 3 parities, such as 1 and 4, is the cube of the correlation between successive lactations. The correlations of milk yield in different parities estimated by Guo et al. [17] did not support the ARH1 residual covariance structure. The model used by Hinde et al. [6] did not fit the animal genetic effect, resulting in their residuals including both animal genetic as well as pure residual effects. The genetic components included in their residuals will create covariance between residuals on related animals. Inappropriately modeling the residual effects by assuming uncorrelated residuals could lead to incorrect conclusions on the effect of calf gender on milk yield. Our study analyzed each parity separately but accounted for correlated residuals due to pedigree. Hinde et al. [6] reported effects in percentage terms, and such values are sensitive to the choice of the mean which can vary according to the model fitted. For example when random animal effects are included, the estimates of fixed effects relate to values that would be observed from founder animals with average EBV of zero.

The model fitted by Hinde et al. [6] did not include contemporary group, although milk production records were pre-adjusted for breed, region, season and parity. The lack of contemporary group may lead to biases in the estimation of calf gender effects, particularly if records on male calves are incomplete for some herds.

Barbat et al. [8] included calf gender in the model used in the French genetic evaluation system [18], which uses a repeatability model that accounted for permanent environmental effects such that the same residual correlation is relevant to any pairs of lactations on the same animal. In a response by the authors of Hinde et al. [6] defending a critical comment by Barbat et al. [8] on their original paper, it was claimed that Barbat’s repeatability model was inappropriate because the effect of first parity calf gender would be picked up by the permanent environmental effect [19]; however, BLUP is translation invariant [20], so when calf gender is fitted as a fixed effect any effect of gender would not be included in the random permanent environmental effect.

This study used an animal model in order to account for all known sources of variation and covariation. Models were fitted for each lactation separately to avoid correlations between residuals and heterogeneous variances in different parities. Fitting a separate model for each lactation is a less powerful approach for modeling milk yield as it does not exploit correlated information from other parities, however it enables the testing of the claim by Hinde et al. [6] that first parity calf gender has an effect on milk yield in later lactations. The analysis of second or third lactation data separately will be affected by selection of cows earlier in their lifetime, however all cows in this study were required to have calf gender records for parities 1–3 and therefore could not have been culled until after their third lactation.

### Single Lactation Milk Yield

Gender of the calf that initiated the lactation and the sequence of calf gender across three parities was shown to have an effect on total lactation yield for lactations 1–3 in New Zealand Holstein Friesian and Jersey cows. Consistent with findings in North American and Iranian Holsteins, giving birth to a daughter was associated with increased milk yield compared to giving birth to a son [6, 7, 9]. Milk yield was between 0.2 and 1.1% higher from a dam with a female compared to a male calf; which was lower than observed in U.S. (~1.3%) and Iranian (~1–2%) studies, but higher than found in the Canadian (~0.4%) study. The French and Danish studies found a small improvement in milk yield (~0.5%) when a male calf was born over a female calf [8, 10].

Hinde et al. [6] observed that first parity calf gender affects second lactation milk yield, however it is also of interest to investigate whether first parity calf gender affects milk yield in later lactations. The analysis of the first three lactations separately in this study allowed a more in-depth analysis of this potential effect. When using the model that did not account for differences in lactation length, first parity calf gender had a significant effect on second parity milk yield in Holstein Friesians but not in Jerseys. First parity gender did not have an effect on second lactation milk yield when lactation length was included in the model. First parity calf gender did not have significant effects on third lactation milk yield in any of the models investigated in this study. It can therefore be concluded that first parity calf gender does not have consistent effects on milk yield in all subsequent lactations in New Zealand Holstein Friesian and Jersey cows.

There are plausible biological reasons to explain differences in first lactation yield due to the gender of the calf that initiated the lactation or the gender of the second calf that was conceived during the first lactation. However, Holstein Friesian cows that gave birth to males in the first three parities had significantly lower first lactation milk yield than cows that gave birth to two males followed by a female in the first three parities when the model did not include lactation length. There are no plausible biological reasons why the gender of the third calf would influence first lactation yield, suggesting that the observed effect of calf gender on milk yield is due to an association between calf gender and milk yield rather than calf gender causing a difference in milk yield. The effect of third parity calf gender on first lactation milk yield was not apparent when lactation length was included in the model, as discussed later.

### Lactation Length

Lactation length was confirmed to be between one and three days shorter when a male calf was born than when a female calf was born, depending on breed and lactation. Although conception date and therefore gestation length was not available on these cows, this finding is consistent with bull calves having longer gestations than female calves by approximately two days [11]. In seasonal calving pastoral farming systems, such as New Zealand, milking ceases for the entire herd on the same day, which introduces a negative relationship between gestation length and lactation length. Milking ceases for the entire herd on the same day due to a process called ‘drying off’ where the farmer takes steps to reduce milk yield so the mammary tissue can rest and regenerate before the next lactation. It is important for the herd to cease on the same day in seasonal calving systems so calving season occurs at the same time every year. Conversely, in North America and other similar dairy systems cows are housed indoors and fed a grain-based diet which means they can have high milk production year round. Under this production system lactation length is typically longer than for seasonal systems because lactation can continue until the milk yield of an individual cow drops to the point that it is more economical to dry her off and remate her. This may lead to a weaker negative correlation between gestation length and lactation length in North American dairy systems. In Iranian Holstein cattle, however, lactation length was observed to be approximately four days shorter following the birth of a male calf compared to a female calf across the first four lactations [9], suggesting that the difference in lactation length in our study may not be restricted to seasonal dairy farming systems.

### Milk Yield with Lactation Length

When a gender-specific lactation length was included in the milk yield model, calf gender did not have an effect on any lactation, except second lactation milk yield in Holstein Friesian cows. When a female calf was born in the second parity, second lactation milk yield was 1.6% higher than when a male calf was born in the second parity. Importantly, third parity calf gender did not have an effect on first lactation milk yield, as was seen when a gender-specific lactation length was not included in the model.

The effect of an additional day of lactation was not significantly different between calf genders in lactations one or three, but was significantly different in lactation two in both breeds, suggesting that lactation two milk yield curves may differ depending on calf gender. The estimated effects of an additional day of lactation during the third lactation showed the greatest difference between genders of any lactation; this difference was not significant due to large standard errors (Table 3). Chegini et. al. [9] evaluated the ratio of lactation in the second and third 100 days of lactation compared to the first 100 days as a measurement of the persistency of lactation and found that cows that gave birth to female calves had more persistent lactations than those that gave birth to male calves, indicating that lactation curves are different between cows that calve male and female calves. Further investigation is necessary to confirm whether the shape of lactation curves differ based on calf gender, and identify potential biological explanations for any such difference.

### Artefacts Present in Total Lactation Milk Yield

Male calves are born, on average, 2 days later than female calves [11]. Herd tests are performed on the same date for the entire herd, so cows that give birth to male calves will have their herd tests performed on average 2 days earlier in the lactation than cows that give birth to female calves. It was found that the interval-centering method does have a 10.8 ± 4.0 L higher milk yield if herd tests are 2 days later in lactation, suggesting that part of the observed difference in calf gender is due to the method used to calculate total lactation milk yield. The observed calf gender difference is too large to be explained only by this difference in herd test dates (Fig 4A).

Cows that calve later in the calving season will typically have a shorter lactation because the entire herd ceases lactation on a single day. When lactation length shortens depending on calving date, as well as the herd tests occurring 2 days earlier, the difference in milk yield is 26.9 ± 6.2 L. This difference is similar to the observed effect of calf gender on milk yield, further supporting that this effect is, at least in part, due to the different lactation length when male calves are born 2 days later (Fig 4B). These results were similar based on Wood’s curves fitted to both Holstein Friesian and Jersey breeds and across lactations.

### Effect on Genetic Gain

Correct modelling of a calf gender-biased milk production could increase genetic gain of milk yield if including calf gender in milk yield prediction models changes the ranking of individuals compared to not including calf gender. In particular, the ranking of the top animals is of greatest importance because it is these animals that go on to become the parents of the next generation. In New Zealand, the largest supplier of premium dairy cattle genetics, LIC, primarily sells straws of semen through teams of the top 30 bulls, with one team per breed. From this team of bulls, a semen straw is randomly chosen to inseminate a cow. The top 30 sires were evaluated to determine whether the top 30 bulls for milk yield would change if calf gender was included in milk yield prediction models. The impact of including calf gender in milk yield evaluations of the top 100 dams was also investigated. More dams were considered to reflect the lower selection intensity applied to females than males in dairy cattle.

Including calf gender in milk yield prediction models resulted in minimal re-ranking of individuals in both Holstein Friesians and Jerseys. In some cases the lowest-ranked bull dropped out of the top 30 sires and some dams ranked below 85^th^ dropped out of the top 100 dams. These re-rankings had little, if any effect on the average EBV of the top 30 sires or 100 dams, suggesting that including calf gender in milk yield prediction models is unlikely to change genetic gain for milk yield in New Zealand Holstein Friesian or Jersey animals.

### Sexed Semen

There have been many studies on the economic benefits of using sexed semen [21, 22], however only one has considered gender-biased milk production in their analysis [23]. Sexed semen exploits a 4% difference in DNA content [24] to reliably produce 90% of offspring of the desired gender [25, 26], but this comes with a drop in fertility with 75–80% the conception rate of conventional frozen-thawed semen [25]. Sexed semen is most commonly used on heifers with high breeding values because this group of animals has the highest fertility rates and need to be producing replacement cows [27]. High fertility rates are an important requirement when using sexed semen because the sorting process damages the sperm to some extent, lowering viability [28].

Assuming gender-biased milk production in favor of female calves results in additional profit from dams that have female calves compared to male calves; therefore, wider use of sexed semen may be economically beneficial. Ettema and Østergaard [23] investigated the effect of gender-biased milk production on milk yield in three scenarios with varying sexed semen use and concluded that including the gender bias increased profitability between €4.0 and €9.9 per cow per year depending on intensity of sexed semen use. The Ettema and Østergaard [23] study used simulated data based on the gender-bias observed in the study by Hinde et al. [6]. The conclusion from the Canadian study into gender-biased milk production was that any increase in milk yield from dams of female calves was not sufficient to warrant the use of sexed semen [7]. The true impact of gender-biased milk production on sexed semen use should be further studied before reliable recommendations can be made into the economic impact of increasing its use.

### Biological Mechanisms

In this New Zealand data from seasonal calving herds, accounting for differences in lactation length was shown to erode the estimates of the effect of calf gender on milk yield. It is possible that gender-biased milk yield can also be explained by other factors that have not been included in the models studied, or have not been adequately defined. Two traits that could explain the observed effect of calf gender on milk yield are birth weight and dystocia.

Milk yield is higher when the calf born is heavier [29] and male calves have been shown to be heavier than female calves [30]; this could lead to the appearance of gender-biased milk production when the increased milk yield is due to males tending to be heavier at birth than females. Chew et al. [31] found that calf gender had no significant effect on total milk, fat or solids when birth weight was included in prediction models, and birth weight had a positive linear relationship with yield traits. Weight is not routinely measured at birth in New Zealand, therefore there were insufficient data to include it in these analyses. Analysis of 174 New Zealand Holstein Friesians with birth weight records showed no effect of either birth weight or calf gender on milk yield, likely due to the low number of individuals and large variation in this trait.

Dystocia, a prolonged or difficult parturition, has been shown to be negatively associated with milk yield [32] and positively associated with birth weight [33]. Male calves are, on average, heavier than female calves [30] and this leads to a higher frequency of dystocia in male calves [33]. This study, as well as the North American and French studies, all dealt with dystocia by removing lactations where the parturition had a calving difficulty score higher than some threshold [6–8], which tended to be whether or not assistance was necessary at birth. Instances of dystocia are often missed, yet the birth process may have been traumatic enough to lower the cow’s milk production. If this is the case then the gender bias found in the North American studies, and in this study, could potentially be due to unidentified or unrecorded dystocia. The effect of dystocia could also explain why calf gender in previous lactations has an effect on milk yield since any birthing complications could leave a lasting physiological effect. A solution to this would be to collect data that provides more accurate classification of dystocia.

Hinde et al. [6] favored a hormone-driven explanation for the effect of calf gender on milk yield whereby a hormone differentially expressed in fetuses of different genders is also differentially expressed in the dam and then affects milk synthesis, possibly through inhibition of mammary gland development. This is a plausible hypothesis, the hormone insulin-like peptide 3 (INSL3) has been shown to be differentially expressed in both fetus and dam, depending on whether the fetus is male or female [34]. This hormone has been shown to affect testicular [4] and ovarian development [35], however it has not yet been shown to directly influence milk yield. Other members of the insulin-like peptide family have been shown to affect mammary development and lactation yield in mice [5]. It is unknown how long it takes for INSL3 to return to base levels after parturition in cattle, therefore it is unknown if it would cause an effect on milk production post-parturition, although it could cause an effect during the lactation when the calf is *in utero*. Although hormones could cause gender-biased milk production, it is more likely to be due to other effects that have been shown to affect milk yield such as birth weight, lactation length and dystocia.

Two other studies have suggested biological explanations for increased milk yield after giving birth to female rather than male offspring, however milk yield was not measured in either study. In humans, mothers of female infants were shown to have upregulation of epithelial/lactocyte genes which may be associated with increased milk yield [36]. A small study in Italian dairy cattle during the transition period observed a difference in nitrogen and energy metabolism between dams that calved males and those that calved females [37]. These studies provide groundwork for further investigating the biological mechanisms behind the effect of calf gender on milk yield.

## Conclusion

This study identified a small effect of calf gender on total milk yield in New Zealand Holstein Friesian and Jersey cows. The effect was present both within parity, with first parity calf gender affecting first lactation milk yield, and across parity, with calf gender sequence across three parities having an effect on a single lactation milk yield. This effect was attributed to the, on average, 2 day increase in gestation length of male calves; causing a shortened lactation in pastoral dairy systems and herd tests being performed on average 2 days earlier throughout lactation. Gender of the calf that initiated the second lactation had an effect on second lactation milk yield in Holstein Friesian cows after accounting for lactation length, indicating that there may be other influences on milk yield that are associated with calf gender, such as birth weight, unidentified dystocia or other physiological changes. The ranking of males and females changed very slightly between a model including and excluding calf gender, indicating that including calf gender is unlikely to impact genetic gain for milk yield in the New Zealand dairy cattle population.

## Supporting Information

S1 Data Milk Yield Data for Lactations 1–3. Columns include animal ID, lactation number, whole lactation milk yield during that lactation, breed, the calf gender sequence over lactations 1–3 or 1–4, number of days in milk during that lactation and the combination of herd the animal was in during that lactation and the year the lactation took place.
(TXT)

S2 Data Pedigree Information for Animals Included in S1 Data. Columns include animal ID, sire ID and dam ID.
(TXT)

S3 Data Herd Test Records for Wood’s Curve Fitting. Columns include animal ID, the date that animal calved and milk yield for up to ten herd test dates, with date (YYYY-MM-DD) as the column heading.
(TXT)

S1 Fig Four-Parity Effect of Calf Gender Sequence on Third Lactation Milk Yield. The four-parity calf gender sequence was fit for 12,347 Holstein Friesian (A) and 4,197 Jersey (B) cows to determine whether first-parity calf gender influenced third lactation milk yield. Producing a male calf rather than a female calf in the first parity did not significantly change third lactation milk yield for any sequence of calf genders in later parities (i.e. paired bars were not significantly different from each other) for either Holstein Friesian or Jersey cows.
(TIF)
