# Supplementary figures and images for: The Effect of Calf Gender on Milk Production in Seasonal Calving Cows and Its Impact on Genetic Evaluations
Source: PLoS One. 2016 Mar 14;11(3):e0151236. doi: 10.1371/journal.pone.0151236 (PMC4790931; doi:10.1371/journal.pone.0151236)

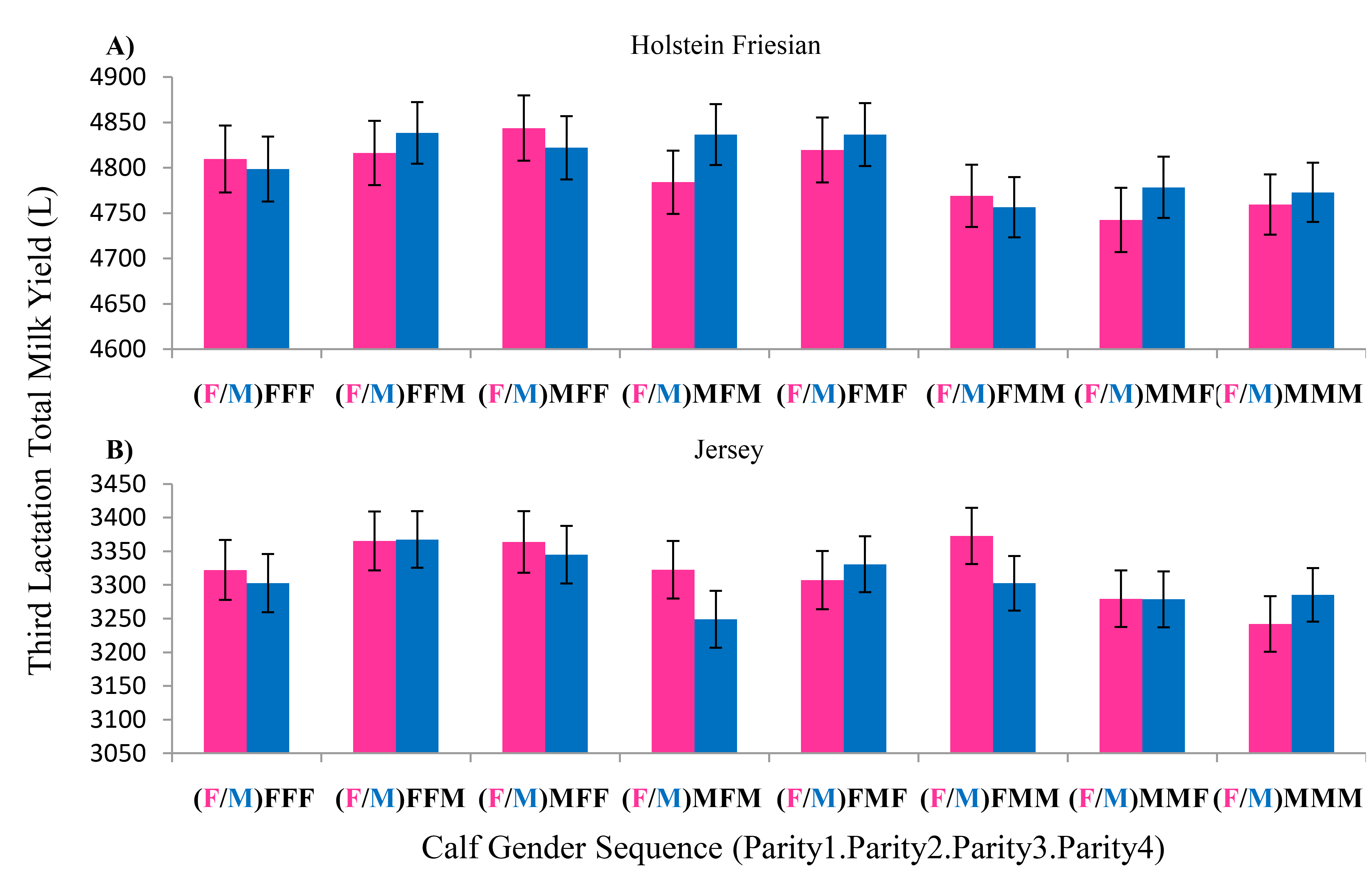

Supplement: S1 Fig — The four-parity calf gender sequence was fit for 12,347 Holstein Friesian (A) and 4,197 Jersey (B) cows to determine whether first-parity calf gender influenced third lactation milk yield. Producing a male calf rather than a female calf in the first parity did not significantly change third lactation milk yield for any sequence of calf genders in later parities (i.e. paired bars were not significantly different from each other) for either Holstein Friesian or Jersey cows. (TIF) [file pone.0151236.s004.tif]
